# Drifting in the abyss: an in-situ observation of swimming in *Psychropotes* (Psychropotidae, Elasipodida, Holothuroidea)

**DOI:** 10.1007/s12526-025-01618-w

**Published:** 2025-12-19

**Authors:** Guadalupe Bribiesca-Contreras, Melanie Mackenzie, Antonina Kremenetskaia, Loïc Van Audenhaege, Bethany F. M. Fleming, Adrian G. Glover, Erik Simon-Lledó, Daniel O. B. Jones

**Affiliations:** 1 Marine Science, National Oceanography Centre, European Way, Southampton, SO14 3ZH UK; 2 Research, Natural History Museum, Cromwell Road, South Kensington, London, SW7 5BD UK; 3 Museums Victoria Research Institute, Museums Victoria, GPO Box 666, Melbourne, VIC 3001 Australia; 4 Nakhimovskiy Pr., Shirshov Institute of Oceanology, Russian Academy of Sciences, 36, Moscow, 117997 Russia; 5 School of Ocean and Earth Science, University of Southampton, Waterfront Campus, European Way, Southampton, SO14 3ZH UK; 6 Institut de Ciències del Mar, ICM-CSIC, Barcelona, 08003 Spain

**Keywords:** Sail-like, Dispersal, ROV, Holothuroid, *Psychropotes semperiana*, Clarion-Clipperton Zone, Sea cucumber, Deep sea, Behaviour

## Abstract

Holothuroids are conspicuous members of megabenthic assemblages, particularly in the deep sea. Some are capable of swimming and while this behaviour was once considered uncommon, deep-sea exploration and technological advances have increased the number of such observations. Swimming or drifting is now regarded to be a potentially common adaptation to deep-sea life that enables long distance dispersal at a low energetic cost and perhaps the ability to track ephemeral food sources. Swimming is well documented for some species, as well as the specialised structures such as brims, swimming lobes, modified podia, and other adaptations like gelatinous consistency, ossicle reduction and flattened bodies. Some species of *Psychropotes* Théel, 1882 have a long ‘tail’ (sail-like unpaired dorsal appendage) that has been suggested to facilitate drifting in near-seabed currents. Yet, to date, swimming had been mainly attributed to early life stages for these species, as these had been collected above the seafloor. While there is evidence that adults can also use this sail-like lobe to drift using bottom currents, the purpose of this dorsal appendage itself is still inconclusive. Here, we document a remotely operated vehicle observation of a long ‘tailed’ adult *Psychropotes* cf. *semperiana* Théel, 1882 actively swimming with the aid of anterior and posterior webbed podia fanning in synchrony, combined with dorso-ventral body flexing, while using the sail-like dorsal appendage to drift in the near-bottom water column.

**Supplementary Information:**

The online version contains supplementary material available at 10.1007/s12526-025-01618-w.

## Introduction

Holothuroids (sea cucumbers) inhabit almost every marine ecosystem, from intertidal to hadal depths, and from the tropics to the poles. They are important components of marine ecosystems and are particularly abundant in the deep sea (Billett 1991; Hansen 1975). They have often been regarded as sedentary animals that move very slowly, with only a few reports of swimming in shallow-water species, such as some members of the genus *Leptosynapta* Verrill 1867 (Costello 1946). However, as early as 1867, Sars observed specimens of the deep-sea species *Bathyplotes natans* (M. Sars 1868) swimming by undulating their entire body when placed in jars after collection. This swimming behaviour was long considered unusual but is now regarded as a relatively common adaptation to the deep sea that allows holothuroids to travel long distances to find suitable habitat or high-quality food at a lower energetic cost (Chimienti et al. 2019; Miller and Pawson 1990; Ohta 1985; Rogacheva et al. 2012).

Two species in the family Pelagothuriidae Ludwig 1893 are well adapted for swimming, with both *Enypniastes eximia* Théel 1882 and *Pelagothuria natatrix* Ludwig 1893 observed to swim by undulating their highly modified anterior veil of fused tube feet or frontal lobe (Gebruk 1995; Miller and Pawson 1990; Ohta 1985; Pawson 1982). The latter species is the only known holothuroid that lacks a benthic life stage*.* Miller and Pawson (1990) noted that approximately 20 species from Synallactidae Ludwig 1894, Psychropotidae Théel 1882, Elpidiidae Thée,l 1882 and Pelagothuriidae, had been found both on and well above the seafloor, and hypothesised that while swimming had only been observed in a small number of bathyal and abyssal species at the time, several additional species had similar morphological characteristics, suggesting they could also be capable of swimming. Increased deep-sea exploration since then has also resulted in numerous reports of swimming holothuroids, being relatively common in members of the family Synallactidae and the order Elasipodida Théel 1882 (Gebruk and Kremenetskaia 2024). Swimming species present external morphological characters such as gelatinous integument and swimming lobes that assist them in swimming (Gebruk and Kremenetskaia 2024).

In the Elasipodida, early life stages have been suggested to be pelagic (Tyler and Billett 1988). Many juveniles from species in the elasipodid family Psychropotidae have been collected in midwater (Billett 1991), with all the smallest specimens—up to 35 mm—only ever collected in pelagic nets (Tyler and Billett 1988). Adults of some species within Psychropotidae have also been observed swimming. *Psychropotes depressa* (Théel 1882) was observed leaping off the seafloor when disturbed, and slowly swimming for short periods of time (Pawson 1976). Similarly, *P. verrucosa* (Ludwig 1893) was observed using contortion followed by a gliding movement when disturbed (Tilot 2006). *Benthodytes lingua* Perrier R., 1896 was observed swimming after collection and trying to escape (Rogacheva et al. 2012).

Some species in the genus *Psychropotes* Théel 1882 present a long, sail-like, unpaired, dorsal appendage—frequently referred to as a ‘sail’ or ‘tail’—that has been suggested to act as a sail and aid in swimming (Gebruk 1995; Mortensen 1927). Juveniles of some species with this long appendage, such as *P. longicauda* Théel 1882, have been collected in midwater trawls at up to 1000 m off the seabed in the northeast Atlantic (Billett et al. 1985), and a juvenile of probably *P. moskalevi* Gebruk & Kremenetskaia in Gebruk et al. 2020 was collected 2000 to 3000 m above the seabed in the Kuril-Kamchatka Trench (Belyaev and Vinogradov 1969). Tilot (1992) disagreed with the premise that *Psychropotes* species with a longer appendage only swim during juvenile stages, having observed footage of a specimen of what appeared to be an adult *P. dyscrita* (Clark 1920) (originally identified as *P. longicauda* but reidentified herein by AK) swimming by rhythmically arching its body. Additionally, the species *P. hyalinus* Pawson 1985 was described from a single adult specimen captured in a trap deployed 5 m above the seafloor in the abyssal Pacific. These observations provided evidence that species of *Psychropotes* with a longer sail-like appendage were likely capable of active swimming as adults, though the purpose of the dorsal appendage itself is still inconclusive.

Here, we present the first recorded observation of an adult *Psychropotes* cf. *semperiana* Théel 1882 actively swimming, while also using the long sail-like appendage to drift with the current. This individual was observed during a remotely operated vehicle (ROV) dive to the abyssal seafloor tracks left by a trial deep-sea mining operation conducted by the Ocean Minerals Company (OMCO) in 1979 (Jones et al. 2025).

## Material and methods

*RRS James Cook* cruise 241 (JC241) to the Clarion-Clipperton Zone (CCZ), eastern Pacific, took place in February–March 2023. It aimed to find and revisit the site of a polymetallic nodule collector test carried out by the Ocean Minerals Company in 1979 to assess recovery 44 years after the disturbance (Jones et al. 2025). A total of 16 imaging transects were conducted using the NOC ROV *Isis* (Marsh et al. 2013). The vehicle was equipped with three cameras: Super Scorpio (https://vocab.nerc.ac.uk/collection/L22/current/TOOL1934/), AESA (http://vocab.nerc.ac.uk/collection/L22/current/TOOL1897/), and a Insite Mini Zeus Mk2 Cam camera (referred to as pilot camera; https://vocab.nerc.ac.uk/collection/L22/current/TOOL1932/). This last was used for navigating and had a fixed position looking forwards, while the other two cameras were in a downward-looking configuration during imaging transects. The ROV *Isis* was also equipped with 4 CATHX Aphos-16 lights and 2 Multi-SeaLite Matrix lights oriented frontward.

## Results and discussion

During the ROV *Isis* Dive 403 (Station JC241-059) on February 28, 2023, at 03:48 UTC, a long ‘tail’ adult *Psychropotes* was observed swimming. The observation lasted about 1 min 45 s and was made in the vicinity of the seafloor tracks left by a deep-sea mining vehicle (Jones et al. 2025), at 13° 43.48′ N, 126° 13.18′ W and a water depth of 4694 m (SM1). Because of the objectives of the dive, it was not possible to change course nor use any of the additional cameras. The lasers were also pointing downwards and out of sight from the pilot camera, preventing a direct and accurate measurement of the specimen.

The specimen had a very distinct long, sail-like, unpaired dorsal appendage, almost as long as the body (c. 0.9 body length), located almost centrally on the dorsum, slightly closer to the posterior end. The body length was estimated at c. 45 cm. That estimate is close to the maximum length reported for this morphotype (40 cm long; Tilot 2006) and to the length of one specimen collected during the same expedition (42 cm at recovery, potentially slightly contracted). Two species of *Psychropotes* with a sail-like appendage have been reported in the CCZ, *P. dyscrita* (Gebruk et al. 2020), and *P.* cf. *semperiana* (Glover et al. 2016). The dorsal appendage of the latter is placed almost centrally on the dorsum; hence the observed specimen was tentatively identified as *P.* cf. *semperiana.* However, the specimen was not collected, and its identity remains uncertain as ossicles could not be examined.

**Fig. 1:**
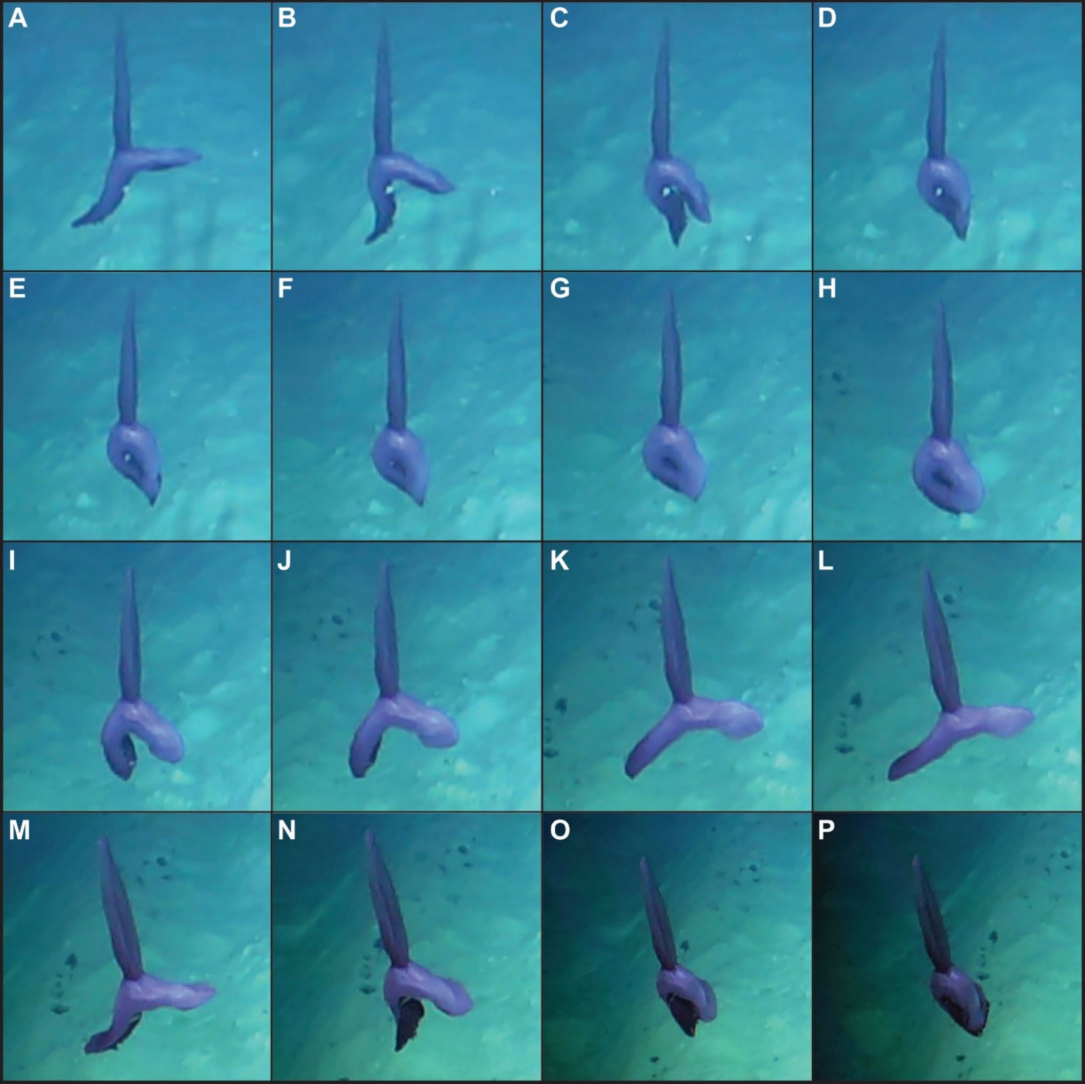
Sequence of active swimming movements in *Psychropotes *cf. *semperiana*. Snapshots taken every 0.4 seconds, every 10 fps from a 25 fps video. Specimen was recorded by the Remotely Operated Vehicle (ROV) Isis during the JC241 expedition on board the RRS James Cook in the Clarion-Clipperton Zone, 13° 43.48' N, 126° 13.18' W, at 4072 m depth. Notice mouth tentacles on **A**–**C**, **M**–**O**. Video credit: The National Oceanography Centre and the Trustees of the Natural History Museum with acknowledgement to the NERC SMARTEX project

The long dorsal appendage may be used as a sail to catch near-seafloor currents. This can be observed from the holothuroid tracking the movement of suspended particles (SM1). The specimen was observed about one metre above the seabed; swimming by dorso-ventral undulation; folding its body in half in the vertical plane (Fig. 1A–F, Fig. 1M–P) and then returning to a horizontal posture (Fig. 1G–L) in a rhythmical motion, while the dorsal appendage remained in a near constant vertical position, acting as a sail. This appendage seemed to also provide additional lift for the animal upwards into the water column. It was observed to drift for up to 4 s between active movements, while in a folded/arched state, and the frequency of the body folding behaviour was around 0.2 Hz. No undulating motion was noted in the dorsal appendage, which remained as an erect sail for the cycle. The specimen also had a marginal brim of fused tube feet around the body, wider anteriorly and posteriorly, forming what seemed like two webbed brims. The body was also flattened anteriorly and posteriorly, thus allowing both webbed podia fans to be used as powerful swimming lobes. These fanned in synchrony, although more force seemed to be exerted by the anterior fan, potentially driving the specimen forwards. This swimming style differs from the metachronal fanning of webbed podia observed in *Enypniastes eximia* (Ohta 1985), but is similar to what has been described for other species lacking a long dorsal appendage, such as *Benthodytes gosarsi* Gebruk 2008 (Rogacheva et al. 2012, 2013), *P. depressa* (Miller and Pawson 1990), and other elongated forms (Gebruk and Kremenetskaia 2024).

Swimming behaviour in holothuroids has been (rarely) observed during surveys in the CCZ, and mostly in the species *Enypniastes eximia* (Tilot 2006), *Peniagone leander* (Bribiesca-Contreras et al. 2022; Pawson and Foell 1986), and *Psychropotes verrucosa* (Ludwig 1893) (Tilot 2006), but not previously for *P.* cf. *semperiana*. Species of *Psychropotes* have been suggested to be occasional swimmers (Gebruk and Kremenetskaia 2024). The observation of a *Psychropotes* specimen contorting itself and gliding when disturbed by the manipulator (Tilot 2006) suggested that swimming could be an escape strategy (Chimienti et al. 2019). However, none of the *P.* cf. *semperiana* specimens collected during the expedition attempted to escape nor seemed disturbed by the bow wave of the ROV, unlike other species (e.g., *Peniagone* spp. and *Benthodytes* spp.) that appeared to escape from the collection box during the ROV dives. *Psychropotes* cf. *semperiana* seems to be widely distributed in the CCZ, spanning over 5,000 km, but is very rare (Simon‐Lledó et al. 2025). Density was estimated at ca. 1 ind/ha (morphotype HOL_044; Simon-Lledó et al. 2023a) from seabed images across eight areas covering over 100,000 m^2^ (Table 1), and only 13 individuals were observed in another three areas from video transects covering ca. 30 km (Simon-Lledó et al. 2023b). Specifically, a single specimen was observed in 3600 m^2^ of seafloor imaged at the OMCO site, with an estimated density of 2.8 ind/ha (Jones et al. 2025).

**Table 1:** Density of *Psychropotes* cf. *semperiana* (morphotype HOL_044) observed in imagery and video transects in the Clarion-Clipperton Zone with details of the different areas surveyed and number of individuals observed. ^*^ Simon-Lledó et al. 2023b; ^**^ Jones et al. 2025

Study area	Area surveyed (m^2^)	HOL_044 (*n*)	Density (ind/ha)
APEI 1^*^	6767	1	1.5
APEI 4^*^	9529	2	2.1
APEI 7^*^	7277	1	1.4
APEI 9^*^	> 8 km	1	
GSR^*^	> 1.2 km	10	
KODOS^*^	> 20 km	2	
NORI-D^*^	5067	2	3.9
TOML_B^*^	24,980	1	0.4
TOML_C^*^	29,275	2	0.7
TOML_D^*^	20,203	2	1.0
OMCO^**^	3600	1	2.8
Total	**106,698**	**12**	**1.1**

The dorsal appendage of all long ‘tail’ *Psychropotes* species may be used in the same way as observed here—a sail that catches the currents. However, the details of active swimming might differ between species depending on the position of the dorsal appendage. The *P.* cf. *semperiana* observed could readily fold in half below the near-central appendage, but other species with a distal appendage might well adopt a different swimming motion. Swimming in *P. dyscrita*, which has an unpaired dorsal appendage located close to the posterior end, was briefly described as an undulating movement that starts in the body and moves to the appendage (Tilot 1992). Based on this observation, Gebruk (1995) suggested that the ‘sail’ can be bent to control movement using bottom currents. In *P. longicauda*—species identification requires confirmation as *P. longicauda* has since been revealed as a cryptic species complex (Gebruk et al. 2020)—the coelomic fluid from the sail-like appendage was found to have significantly lower SO_4_^2−^ ions concentrations with respect to the surrounding seawater which slightly reduced the fluid density in the chambers, thereby imparting some degree of buoyancy in the ‘tail’ (L. Hawkins, pers. comm.). This ionic regulation has been found in many planktonic invertebrates and has been suggested as the most energetically economical buoyancy mechanism (Newton and Potts 1993). Swimming could also contribute to the widespread distribution of *P. semperiana.* This behaviour has been regarded as an important mechanism for long distance dispersal (Ohta 1985), and possibly allows them to reach environments that are inaccessible to other deposit feeders incapable of swimming, as suggested for *Penilpidia ludwigi* (Marenzeller 1893) (Chimienti et al. 2019). Further studies assessing connectivity patterns, life-history traits, and evolutionary histories are needed to understand the role that swimming has on connectivity, distributional ranges, and habitat exploitation.

## Supplementary Information

Below is the link to the electronic supplementary material.

ESM 1 (MP4 34.7 MB)

## Declarations

### Data availability

All data relevant to this study have been included in the main text or as Supplementary Material.

## References

[CR1] Belyaev GM, Vinogradov ME (1969) A new pelagic holothurian (Elasipoda, Psychropotidae) from abyssal depths in the Kurile-Kamchatka Trench. Zool Zh 48:709–716

[CR2] Billett DSM (1991) Deep-sea holothurians. Oceanogr Mar Biol 29:259–317

[CR3] Billett DSM, Hansen B, Huggett QJ (1985) Pelagic Holothurioidea (Echinodermata) of the Northeast Atlantic. In: Keegan BF, O’Connor DS (eds) Echinodermata. CRC Press, pp 399e411

[CR4] Bribiesca-Contreras G, Dahlgren TG, Amon DJ, Cairns S, Drennan R, Durden JM, Eleaume MP, Hosie AM, Kremenetskaia A, McQuaid K, O’Hara TD, Rabone M, Simon-Lledo E, Smith CR, Watling L, Wiklund H, Glover AG (2022) Benthic megafauna of the western Clarion-Clipperton Zone, Pacific Ocean. Zookeys 1113:1–110. 10.3897/zookeys.1113.82172 36762231 10.3897/zookeys.1113.82172 PMC9848802

[CR5] Chimienti G, Aguilar R, Gebruk AV, Mastrototaro F (2019) Distribution and swimming ability of the deep-sea holothuroid *Penilpidia ludwigi* (Holothuroidea: Elasipodida: Elpidiidae). Mar Biodivers 49:2369–2380. 10.1007/s12526-019-00973-9

[CR6] Clark AH (1920) Echinoderms. Report of the Canadian Arctic Expedition 1913–1918. Vol. VIII. Mollusks, Echinoderms, Coelenterates, etc. Part C: Echinoderms: 1c-13c

[CR7] Costello DP (1946) The swimming of *Leptosynapta*. Biol Bull 90:93–96

[CR8] Marenzeller Ev (1893) Berichte der Commission für Erforschung des östlichen Mittelmeeres. Zoologische Ergebnisse. 1.Echinodermen gesammelt 1890, 1891 und 1892. Denkschr Kaiserl Akad Wiss

[CR9] Gebruk AV (1995) Locomotory organs in the elasipodid holothurians: functional-morphological and evolutionary approaches. In: Emson R, Smith A, Campbell A (eds) Echinoderm research. Balkema, A. A, pp 95–102

[CR10] Gebruk AV (2008) Holothurians (Holothuroidea, Echinodermata) of the northern Mid-Atlantic Ridge collected by theG.O. SarsMAR-ECO expedition with descriptions of four new species. Mar Biol Res 4:48–60. 10.1080/17451000701842898

[CR11] Gebruk A, Kremenetskaia A (2024) Swimming sea cucumbers. In: Mercier A, Hamel J-F, Suhrbier AD, Pearce CM (eds) The world of sea cucumbers. Academic Press, pp 351–359

[CR12] Gebruk AV, Kremenetskaia A, Rouse GW (2020) A group of species “*Psychropotes longicauda*” (Psychropotidae, Elasipodida, Holothuroidea) from the Kuril-Kamchatka Trench area (North-West Pacific). Prog Oceanogr 180 10.1016/j.pocean.2019.102222

[CR13] Glover AG, Wiklund H, Rabone M, Amon DJ, Smith CR, O’Hara T, Mah CL, Dahlgren TG (2016) Abyssal fauna of the UK-1 polymetallic nodule exploration claim, Clarion-Clipperton Zone, central Pacific Ocean: Echinodermata. BDJ: e7251 10.3897/BDJ.4.e7251 10.3897/BDJ.4.e7251 PMC4759440 26929713

[CR14] Hansen B (1975) Systematics and biology of the deep-sea Holothurians: 1. Elasipoda. Scandinavian Science Press, Copenhagen

[CR15] Jones DOB, Arias MB, Van Audenhaege L, Blackbird S, Boolukos C, Bribiesca-Contreras G, Copley JT, Dale A, Evans S, Fleming BFM, Gates AR, Grant H, Hartl MGJ, Huvenne VAI, Jeffreys RM, Josso P, King LD, Simon-Lledo E, Le Bas T, Norman L, O’Malley B, Peacock T, Shimmield T, Stewart ECD, Sweetman AK, Wardell C, Aleynik D, Glover AG (2025) Long-term impact and biological recovery in a deep-sea mining track. Nature. 10.1038/s41586-025-08921-3 40139245 10.1038/s41586-025-08921-3 PMC12137123

[CR16] Ludwig H (1893) Vorläufiger Bericht über die erbeuteten Holothurien. Bulletin of the Museum of Comparative Zoöology at Harvard College Reports on the Dredging Operations off the West Coast of Central America to the Galapagos, etc., by the U. S. Fish Commission Steamer “Albatross”. IV.: 105–114

[CR17] Ludwig H (1894) The Holothurioidea. In: Reports on an exploration off the west coasts of Mexico, Central and South America, and off the Galapagos Islands, in charge of Alexander Agassiz, by the U. S. Fish Commission Steamer “Albatross,” during 1891, Lieut. Commander Z. L. Tanner, U. S. N., commanding. XII. Memoirs of the Museum of Comparative Zoölogy at Harvard College 17: 183 pp

[CR18] Marsh L, Copley JT, Huvenne VAI, Tyler PA, the Isis ROV Facility (2013) Getting the bigger picture: using precision remotely operated vehicle (ROV) videography to acquire high-definition mosaic images of newly discovered hydrothermal vents in the Southern Ocean. Deep-Sea Res II 92:124–135. 10.1016/j.dsr2.2013.02.007

[CR19] Miller JE, Pawson DL (1990) Swimming sea cucumbers (Echinodermata: Holothuroidea): a survey, with analysis of swimming behavior in four bathyal species. Smithson Contrib Mar Sci 35:1–18

[CR20] Mortensen T (1927) Handbook of the echinoderms of the British Isles. Oxford University Press, London

[CR21] Newton C, Potts WTW (1993) Ionic regulation and buoyancy in some planktonic organisms. J Mar Biol Assoc U K 73:15–23

[CR22] Ohta S (1985) Photographic observations of the swimming behavior of the deep-sea pelagothuriid holothurian *Enypniastes* (Elasipodida, Holothuroidea). J Oceanogr Soc Japan 41:121–133

[CR23] Pawson DL (1976) Some aspects of the biology of deep-sea echinoderms. Thalassia Jugosl 12:287–293

[CR24] Pawson DL (1982) Papers from the echinoderm conference. 8. Deep-sea echinoderms in the Tongue of the Ocean, Bahama Islands: a survey, using the research submersible Alvin. Aust Mus Mem 16:129–145. 10.3853/j.0067-1967.16.1982.362

[CR25] Pawson DL (1985) *Psychropotes hyalinus*, new species, a swimming elasipod sea cucumber (Echinodermata: Holothuroidea) from the north central Pacific Ocean. Proc Biol Soc Wash 98:523–525

[CR26] Pawson DL, Foell EJ (1986) *Peniagone leander* new species, an abyssal benthopelagic sea cucumber (Echinodermata: Holothuroidea) from the eastern central Pacific Ocean. Bull Mar Sci 38:293–299

[CR27] Perrier R (1896) Sur les Élasipodes recueillis par le Travailleur et le Talisman. CR Hebd Seances Acad Sci 123:900–903

[CR28] Rogacheva A, Gebruk A, Alt CHS (2012) Swimming deep-sea holothurians (Echinodermata: Holothuroidea) on the northern Mid-Atlantic Ridge. Zoosymposia 7:213–224. 10.11646/zoosymposia.7.1.19

[CR29] Rogacheva A, Gebruk A, Alt CHS (2013) Holothuroidea of the Charlie Gibbs Fracture Zone area, northern Mid-Atlantic Ridge. Mar Biol Res 9:587–623. 10.1080/17451000.2012.750428

[CR30] Sars M (1868) Om Afbildninger af nogle af hans son I forrige Aar ved Lofoten fundne Echinodermer og Coelenterater. Forh Vidensk-Selsk Kristiania 1867:19–23

[CR31] Simon-Lledó E, Amon DJ, Bribiesca-Contreras G, Cuvelier D, Durden JM, Ramalho SP, Uhlenkott K, Arbizu PM, Benoist N, Copley J, Dahlgren TG, Glover AG, Fleming B, Horton T, Ju SJ, Mejia-Saenz A, McQuaid K, Pape E, Park C, Smith CR, Jones DOB (2023b) Carbonate compensation depth drives abyssal biogeography in the northeast Pacific. Nat Ecol Evol 7:1388–1397. 10.1038/s41559-023-02122-9 37488225 10.1038/s41559-023-02122-9 PMC10482686

[CR32] Simon‐Lledó E, Baselga A, Gómez‐Rodríguez C, Metaxas A, Amon DJ, Bribiesca‐Contreras G, Durden JM, Fleming B, Mejía‐Saenz A, Taboada S, Van Audenhaege L, Jones DOB (2025) Marked variability in distance‐decay patterns suggests contrasting dispersal ability in abyssal taxa. Glob Ecol Biogeogr. 10.1111/geb.13956

[CR33] Simon-Lledó E, Amon DBC, Guadalupe, Cuvelier D, Durden JM, Ramalho SP, Uhlenkott K, Martinez Arbizu P, Benoist N, Copley J, Dahlgren TG, Glover AG, Fleming B, Horton T, Ju S-J, Mejia-Saenz A, McQuaid K, Pape E, Park C, Smith CR, Jones DOB (2023a) Abyssal Pacific Seafloor Megafauna Atlas (1.0) 10.5281/zenodo.8172728

[CR34] Théel H (1882) Report on the Holothuroidea dredged by H.M.S. ‘Challenger’ during the years 1873–76. Part i. Report on the scientific results of the voyage of H.M.S. Challenger during the years 1873–1876. Zoology 4:1–176

[CR35] Tilot V (1992) La structure des assemblages mégabenthiques d’une province à nodules polymétalliques de l’océan Pacifique tropical est

[CR36] Tilot V (2006) Biodiversity and distribution of the megafauna. Vol 2. Annotated photographic atlas of the echinoderms of the Clarion-Clipperton Fracture Zone. Intergovernmental Oceanographic Commission Technical Series 69

[CR37] Tyler PA, Billett DSM (1988) The reproductive ecology of elasipodid holothurians from the N. E. Atlantic. Biol Oceanogr 5:273–296. 10.1080/01965581.1987.10749518

[CR38] Verrill AE (1867) Notes on Radiata in the Museum of Yale College, with descriptions of new genera and species. No. 3. On the geographical distribution of the echinoderms of the West coast of America. Trans Conn Acad 1:323–351

